# Assessing dependence between frequency and severity through shared random effects

**DOI:** 10.1371/journal.pone.0271904

**Published:** 2022-08-19

**Authors:** Devan G. Becker, Douglas G. Woolford, Charmaine B. Dean

**Affiliations:** 1 Department of Statistical and Actuarial Sciences, The University of Western Ontario, London, Ontario, Canada; 2 Department of Statistics and Actuarial Sciences, The University of Waterloo, Waterloo, Ontario, Canada; Universita degli Studi di Napoli Federico II, ITALY

## Abstract

Research on the occurrence and the final size of wildland fires typically models these two events as two separate processes. In this work, we develop and apply a compound process framework for jointly modelling the frequency and the severity of wildland fires. Separate modelling structures for the frequency and the size of fires are linked through a shared random effect. This allows us to fit an appropriate model for frequency and an appropriate model for size of fires while still having a method to estimate the direction and strength of the relationship (e.g., whether days with more fires are associated with days with large fires). The joint estimation of this random effect shares information between the models without assuming a causal structure. We explore spatial and temporal autocorrelation of the random effects to identify additional variation not explained by the inclusion of weather related covariates. The dependence between frequency and size of lightning-caused fires is found to be negative, indicating that an increase in the number of expected fires is associated with a decrease in the expected size of those fires, possibly due to the rainy conditions necessary for an increase in lightning. Person-caused fires were found to be positively dependent, possibly due to dry weather increasing human activity as well as the amount of dry few. For a test for independence, we perform a power study and find that simply checking whether zero is in the credible interval of the posterior of the linking parameter is as powerful as more complicated tests.

## 1 Introduction

Wildland fire managers use the output of complex statistical calculations to guide resource management decisions. There are a wide variety of models in the literature for modelling the number of fires and their size [1]. However, these two outcomes are typically modelled independently; that is, the marks are treated as separable from the points. One might assume, however, the probability of ignition (frequency) should be associated with the size of the fires (severity) since landscapes or weather conditions that are more conducive to ignitions would also be conducive to larger fires. Ignoring this correlation may lead to systematically biased models. Our aim in this study is to characterize the dependence between ignitions and size and develop a test for dependence between size and count in a compound model.

Correlation between the two outcomes can be either positive or negative. Positive correlation would imply that the same conditions that lead to an increase in frequency also lead to an increase in severity. Negative correlation implies that there are either many small fires or a few large fires. This may be due to small fires clearing the landscape and therefore not allowing large fires to burn (see, e.g., [2]). This latter case applies over the course of a season; the correlation between frequency and severity is assumed to be a property of the data over an entire year rather than for estimates on a single day. This interpretation provides insights into long-term climate and weather effects.

A common method for jointly modelling two outcomes is to use shared random effects. This is commonly used in joint longitudinal and time-to-event models, where the longitudinal outcomes and the survival times of the subjects are modelled conditional on a shared random effect (or frailty) that is unique to each subject [3]. [4] use Bayesian methods to model several distributions jointly with a shared random effect. [5] employed this framework to model a count distribution jointly with the severity of events, which is similar to what we do here.

Poisson-type distributions are often used in modelling the daily counts of wildland fires. [6] used a binomial distribution to predict the probability of a fire day, namely the probability that a given location at a given point in time will have at least one fire. This technique approximates a Poisson distribution under the assumption that the probability of more than one fire in a small spatial area is negligable. [7] used a Poisson model for the daily counts of person-caused fires based on a measure of fuel moisture (Fine Fuel Moisture Code). Similarly, [8] used a negative binomial distribution to model the daily count of bushfires in Australia and [9] used a zero-inflated negative binomial distribution with spline terms for wildland fires in California.

Instead of modelling counts of the number of fires, many authors [10–13] use a discretized approach, partitioning the study area and period into a set of fine-scale space-time cells (commonly referred to as voxels). Then, counts are mapped to a presence/absence (i.e., 1/0) response variable which can be modelled using classification methods. For example, [10] partitioned a portion of Oregon into cells and used a logistic regression to model the probability of a fire within a given cell. They note that this is an approximation to a spatio-temporal Poisson process with an inhomogeneous intensity function that depends on other covariates. In such models it is common to include a seasonality component. For example, [14] demonstrated how such seasonal trends can vary spatially and by ignition cause (lightning vs. person-caused) over a large area of Ontario’s former Intensive Fire Management Zone. [15] used Log-Gaussian Cox Process models to estimate the number of fires in a given cell. These models estimated the point process intensity in each pixel of a map based on spatial covariates such as elevation, distance to urban areas, and land use. They used a zero-inflated Poisson model (a Poisson model with a process leading to excess zeroes) as well as a hurdle Poisson model (a strictly positive Poisson model with a separate process leading to zeroes) model for the count within each cell.

Fire sizes have also been investigated by many researchers but there is little agreement on appropriate parametric models. [16] provided estimates for several different parametric distributions for fire sizes in Los Angeles, California, including Pareto, exponential, and lognormal. The authors concluded that a truncated or tapered Pareto model was the best fit. [17] found that a truncated exponential distribution with covariates was an excellent fit to their data, which was lightning-caused fires in Alberta. [18] determined that a Pareto model (i.e. power-law distribution) was appropriate for four data sets from the continental United States, Alaska, and Australia, although later authors dispute this claim, as discussed below.

Very little research has explored the relationship between fire size and count. [19] used a compound Poisson model for the total area burned (i.e., the random sum of random fire sizes). This was done using moment-based estimates of the fire size distribution, which provided easily interpretable results but does not allow for the inclusion of covariates for the size distribution (however, the count distribution could incorporate covariates). Podur also looked at statistical quality control charts for the mean and variance of size and count of fires, but these control charts all treated the count and the size as independent. [9] estimated a zero-inflated negative binomial distribution for counts and a lognormal distribution for fire sizes, but these models were also treated as independent.

[18] concluded that the joint distribution of the size and count could be characterized as a power-law distribution, from which they infer that, similar to earthquakes, a lack of small and medium sized fires is associated with an increased probability of large fires. [20] reexamined deviations from this power-law behaviour using a birth-death process for ignition and extinguishment, concluding that power-law behaviour would only hold if both ignition and extinguishment relied on the size of the fire in the same way, which they assert is unlikely. Even with deviations from power-law behaviour, these conclusions are consistent with the idea that fire count and size are negatively correlated.

[21] investigated the dependence between estimates of the point process intensity and mark distribution (i.e., distribution of fire sizes) over time by treating them as marked spatio-temporal point processes. Non-parametric tests based on spatial versions of the Cramer von Mises and Kolmogorov-Smirnov tests were applied to forest fires in Los Angeles, California. These tests invloved estimating the joint distribution and the product of the marginal distributions and testing for a difference in the resulting estimates. Schoenberg concluded that the quantities were dependent.

Forest fires can be classified as lightning-caused or person-caused, where person-caused includes recreational activities (e.g., camping or hiking) and industrial activity (e.g., forestry or sparks from railways), among other causes. Previous studies have shown that person-caused and lightning-caused fires have fundamentally different distributions. For instance, [22] found that the spatial distribution, ignition conditions, lengths, and sizes of fires in peninsular Spain are significantly different between the two causes. Similar conclusions are discussed in [23, 24]. [25] found that suppression efforts can be different when the fire managers know the cause of the fire.

Because of these differences, it is commonplace to fit models to lighting-caused or person-caused fires separately. This is demonstrated in, e.g., [2, 6–8, 26–28], who all specified the cause of the fires that they study in the title of their papers.

In our analysis of the size and frequency, we build on the approaches mentioned above with a specific focus on jointly modelling frequency (i.e., counts of fire occurrences) and severity (i.e., size of fires). To model the counts, we use a negative binomial model. We include a hurdle component as our counts appear to have excess zeros. The fire sizes are modelled with a lognormal distribution, but with considerations for the unique features of our data: the data are rounded to the nearest 0.1 hectares with preference to multiples of 0.5, and a large portion of our data is exactly 0.1 hectares.

[5] fit a model that modelled the dependence between count and severity for panel counts in hormone therapy treatments. They had three response variables: an indicator for presence of an event (hot flash) on a given day, a zero-inflated Poisson count of number of events, and an indicator for whether the patient had a high-severity day.

Similar to [5], we model the dependence between count and size with a shared random effects framework. This allows us to specify a model for frequency and a model for severity, then link these models with a random effect term. We include a linking parameter on the random effect. This parameter accounts for a difference in effect size for the random effect given that the responses have different scales, with the consequence of providing a test for independence between the two outcomes. Our random effects structure, our focus on the tests for independence, and the considerations for our data distinguish our approach from [5].

## 2 Data and study area

We apply our models to fire occurrence data from 1953 to 1995 in the Canadian province of British Columbia (BC). For each individual fire, we have the coordinates of the estimated ignition point as well as key time points in the lifetime of each fire, such as the time it was ignited (often estimated), reported to the management agency, declared “being held” (defined as a fire that is still being fought but is not expected to grow), and declared “out,” along with the approximate size at each of these times. Also recorded are weather variables, such as temperature, wind, humidity, etc., at the report date of each fire and a measure of the fuel build-up and fire weather, as defined by [29, 30]. We treat lightning-caused fires and person-caused fires as two separate types of fires, and the models described in the paper are applied to each cause independently.

**Fig 1:**
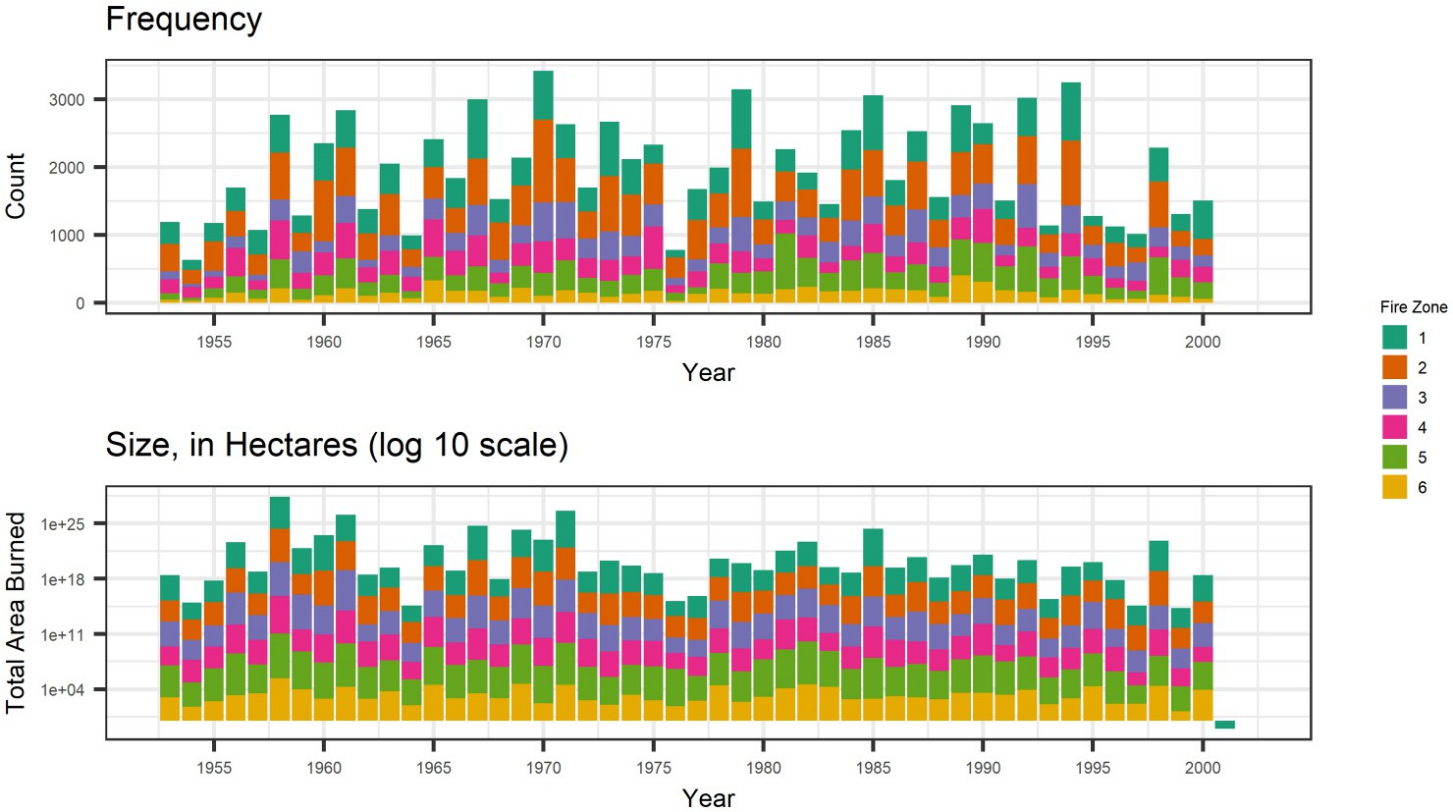
The number and size of fires in each zone, coloured by fire zone. The number of fires is equally distributed across regions, but region 5 tends to have much larger fires than the other regions.

We focus on the daily counts of fires (using the ignition time to determine the date) and the sizes of those fires on a given day. Due to potential changes to fire management strategies over time and differences in weather from year to year, we do not have a consistent definition of the operational fire season. We chose April 1st to November 1st as our fire season to reduce the number of zero fire days while not rejecting too many fires. Less than 1% of our fires are removed by this definition. There is evidence that the length of the fire season is increasing with the changing climate [31, 32], but we use the same definition for all years for our model building. The total numbers and sizes of fires for each year in each zone during our chosen fire season are shown in Fig 1.

British Columbia is partitioned into six fire management zones, which we will refer to generally as regions. The regions are defined in the data set and can be viewed on a map at www2.gov.bc.ca (the copyright terms do not allow the maps to be included in this publication). These regions are used both for topological and administrative reasons. For an example of the differences between regions, regions 5 and 6 (Prince George and Northwest) are separated by the Coastal Mountain Range, whereas regions 2 and 3 (Kamloops and Caribou) are separated by a highway with Kelowna on one side and Kamloops on the other. Region 1 (Southeast) is comprised mostly of mountains, valleys, and rivers with most of the population along the highway to Kelowna, and Region 4 (Coastal) contains Vancouver, Vancouver Island, and the coastal mountain ranges. Although these regions are quite large, we assume that they are relatively homogeneous in terms of vegetation cover and climate.

For both the negative binomial model for counts and the lognormal fire sizes, it is straightforward to include covariates in the respective mean parameters. For fire sizes, these covariates are based on recorded weather variables as well as variables defined by the Canadian Forest Fire Danger Rating System [28]. These covariates take into account the temperature, wind, amount of fuel, moisture, and recent precipitation on the day of the fire’s ignition.

The choice of covariates for the count distribution is nontrivial. We require a covariate that influences the number of fires over a broad region that changes throughout the year, otherwise it would not be descriptive of changes in expected daily numbers of fires. We downloaded data from all weather stations in B.C. (http://climate.weather.gc.ca) that had daily observations of precipitation (minimum, maximum, and mean), wind (maximum gust speed), and temperatures (minimum, maximum, and mean) between 1953 and 1995. In total, 107 stations were included. For each day, the data were averaged across all stations within a region. The daily average of the measurement in a given region acts as our covariate.

Some features of our data require extra consideration. First, the numbers of wildfires in our data have an excess of zeroes. This is overcomefg with a hurdle model. The hurdle component in our modelling framework means that there is some probability of seeing 0 fires in a given day and a complementary probability of a positive count.

The fire sizes in our data set are all rounded to the nearest 0.1 hectares (ha). Marginal estimates of a lognormal distribution as a generalized linear model indicate that the mean on the log scale is less than 0, which leads to non-ignorable rounding effects. This is further complicated by digit preference, where multiples of 0.5 are preferred over neighbouring values over a study period where the units of measurement changed from the imperial system to the metric system. This is yet further complicated by the fact that fires with a size of exactly 0.1 ha make up approximately 60% of our data.

**Fig 2:**
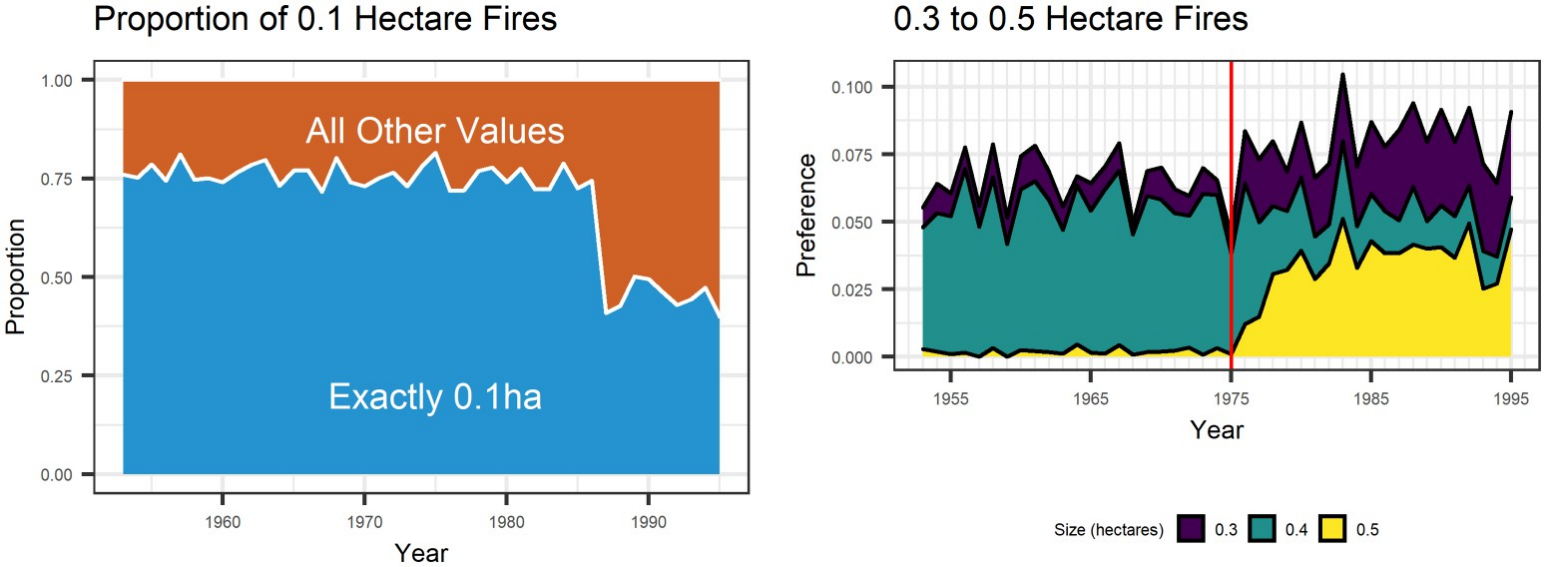
Evidence of rounding and unit change. Prior to 1985, 75% of all fires were recorded as being exactly 0.1ha. The decrease in proportion coincides with the advent of fires recorded as 0ha. In 1975, Canada switched to the metric system. This means that fires were approximated as being 0.5ha rather tham 0.4ha (1 acre). Note that the proportion of 0.1ha fires did not change since 1/10th of a hectare and 1/4th of an acre are both coarsening values.

Fig 2 demonstrates these coarsening issues. The recorded distribution of fire sizes has changed drastically over time, but this is likely an artifact of the measurements. In approximately 1975, the fire managers switched from metric to imperial units. Since 1 hectare ≈ 0.4 acres (ac), this change can be seen in the proportion of fires recorded as a multiple of 0.4 ha (including multiples of 0.2 ha, which is 0.5 ac).

A measurement of 0.1 ha is a preferred number regardless of the unit system (0.1 ha ≈ 1/4 ac), so this did not change in the switch from imperial to metric units. However, the number of 0.1 ha fires decreased suddenly in 1987. Also in 1987, we see the first time where fires were recorded as 0 ha, which was only seen a few times in 1953 to 1986. It may be the case that 0 ha fires recorded after 1987 would have been recorded as 0.1 ha had they occurred prior to 1987.

## 3 Methods

### 3.1 Model framework

Let *π*_*ir*_ represent the probability that the number of fires on day *i* in region *r* is larger than 0, and hence 1 − *π*_*ir*_ denotes the probability that this count is 0. To model the covariate effects, a variety of forms of *π*_*ir*_ are possible; here we use logistic regression. The number of positive counts is modeled as a negative binomial distribution truncated at 0, with a mean that is dependent on covariates as well as a day-specific random effect *b*_*i*_. Let *N*_*ir*_ be the number of fires on day *i* in region *r*. Then, we have:

$$\begin{array}{cc} P(N_{ir}=n|\theta^{\left(\pi \right)},\theta^{\left(N\right)},{Z_{ir}}^{\left(N\right)},b_{i}) & =\{\begin{array}{cc} \left(1-\pi_{ir}\right) & \text{if}\;n=0\\ \pi_{ir}P\left({N_{ir}}^{*}=n\right) & \text{if}\;n\geq 1 \end{array},\\ {N_{ir}}^{*} & ∼\text{Truncated}\;\text{Negative}\;\text{Binomial}(\lambda_{ir},\alpha_{i}),\\ P({N_{ir}}^{*}=n|\theta^{\left(N\right)},{Z_{ir}}^{\left(N\right)},b_{i}) & =\frac{\Gamma (n+\alpha_{i}^{-1})}{\Gamma \left(\alpha_{i}^{-1}\right)\Gamma \left(n+1\right)}{\left(\frac{1}{1+\alpha_{i}\lambda_{ir}}\right)}^{\alpha_{i}^{-1}}{\left(\frac{\alpha_{i}\lambda_{ir}}{1+\alpha_{i}\lambda_{ir}}\right)}^{n} \end{array}$$ (1)

where *θ*^(*π*)^ refers to the vector of parameters that are exclusive to the hurdle component of the model, *θ*^(*N*)^ refers to the parameters for the negative binomial model, $Z_{ir}^{\left(N\right)}$ refers to the covariates for the negative binomial model that were recorded on day *i* in region *r* with coefficient vector *β*^(*N*)^, λ_*ir*_ is the mean, and *α*_*i*_ is the dispersion of the truncated negative binomial distribution.

The parameters are modelled as follows:

$$\begin{array}{cc} \text{log}(\lambda_{ir}) & =t_{\lambda }\left(i\right)+\lambda_{r}+\beta^{\left(N\right)}{Z_{ir}}^{\left(N\right)}+b_{i},\\ \text{logit}(\pi_{ir}) & =t_{\pi ,r}\left(i\right),\\ \text{log}(\alpha_{i}) & =t_{\alpha }\left(i\right) \end{array}$$ (2)

where *t*_λ_(*i*), *t*_*π*,*r*_(*i*), and *t*_*α*_(*i*) are temporal trends, here modelled as P-splines with cubic basis functions [33] and knots defined at the deciles of the fire season. The knots were chosen under the assumption that the parameters have the same smoothness throughout the season. For further information about spline-based smoothing the interested reader is directed to the books of [34, 35] and references therein.

The sizes of the fires are modelled as lognormal. However, fire sizes are only recorded to the first decimal place. Approximately 90% of the fires are less than one hectare, so this coarsening can cause dramatic bias in the results [36]. To account for this, we make assumptions about the rounding behaviour. We treat all of our data as interval censored, with intervals chosen to reflect our fire science context. We discuss the model for the distribution of fire sizes next, and in Section 4.2 of this paper we consider in detail how interval censoring of the fire sizes is handled.

Let *X*_ijr_* represent the true fire size and *X*_*ijr*_ represent the observed fire size, then:

$${X_{ijr}}^{*}∼\text{LogNormal}(\mu_{ijr},\sigma_{r}^{2}),$$ (3)

$$\mu_{ijr}=\mu_{r}+\beta^{\left(X\right)}Z_{ijr}^{\left(X\right)}+\gamma_{r}b_{i}$$ (4)

The covariates *Z*_*ijr*_^(*X*)^ were mean-centred for better convergence properties in the Bayesian Markov Chain Monte Carlo (MCMC) algorithm [37].

The shared random effect *b*_*i*_ is constant across the province but different for each day of the fire season. We may model the random effect in a variety of ways; for example with either independent Gaussian distributions or with an autoregressive (AR) structure to account for temporal autocorrelation:

$$\begin{array}{cc} b_{i} & ∼N(0,{\sigma_{b}}^{2})\text{,}\;\text{or}\\ b_{0} & =0;b_{i}∼N\left(\phi b_{i-1},{\sigma_{b}}^{2}\right)\text{,}\;\text{or}\\ b_{-1} & =0;\;b_{0}=0;\;b_{i}∼N\left(\phi_{1}b_{i-1}+\phi_{2}b_{i-2},{\sigma_{b}}^{2}\right) \end{array}$$ (5)

The linking parameter *γ*_*r*_ is different for each region. This is required in the model for the size distribution to account for the differences in scales of the two outcomes (count and size). For instance, *b*_*i*_ = 1 corresponds to an increase in *e*^1^ times as many fires in the count distribution, but an increase of *γ*_*r*_ in the mean of the size distribution. By the including this shared random effect, the model incorporates correlation between the two outcomes. Table 1 identifies how the shared random effect links the two outcomes, and the effect of the correlations.

**Table 1:** Interpretations of the linking parameter.

	*b*_*i*_ > 0	*b*_*i*_ < 0
*γ*_*r*_ > 0	Large fires, Many fires	Small, Few
*γ*_*r*_ < 0	Small, Many	Large, Few

### 3.2 Models for rounded and heaped fire sizes

We consider three possibilities for the true fire sizes: (M1) the data were rounded to the nearest 0.1; (M2) most of the data were rounded to the nearest 0.1 but some were preferentially rounded to either 0.1, 0.5, 1, 1.5, or 2; or (M3) a probabilistic mixture of these two. The mixture model allows for a probability that it was rounded to either level of precision at the expense of added complexity. M1 will be referred to as rounding, M2 will be referred to as heaping, and M3 will be referred to as the mixture model.

Models M1 and M2 are achieved by treating the data as interval censored. The same estimation algorithm is used to fit both models, but different definitions of intervals are used.

Model M3, the mixture model, is based on [38]. We denote the rounding regime as *G*_*ijr*_, with regime *G*_*ijr*_ = 1 corresponding to rounding to the nearest decimal place and *G*_*ijr*_ = 2 corresponding to rounding to the nearest 0.5. For example, a recorded value of 0.5 may correspond to a true value between 0.45 to 0.55 (M1) or a true value between 0.25 and 0.75 (M2). Fires recorded as 0.1 ha are a special case and are treated as if they are a multiple of 0.5, i.e. their interval is 0.1 ± 0.25, truncated at 0.

The rounding intervals given are measured in hectares. However, as previously stated it is evident that the data collection was completed using the imperial system prior to 1975 and the metric system after (Fig 2). With the unit change came a change in digit preference. For data before 1975, M2 relates to multiples of 1 acre; for instance, rounding to the nearest 0.5 means nearest 0.5 ac, which is recorded in our data as 0.2 ha. Rounding to the nearest 0.1 ha remains the same regardless of the measurement units, since 0.1 ha is approximately 0.25 ac.

Let *X*_*ijr*_ represent the observed fire size (subject to rounding and/or coarsening), then:

$$\begin{array}{cc} F(x) & =\int_{-\infty }^{x}f_{X^{*}}\left(t|\sigma_{xr},\mu_{ijr},b_{i}\right)dt\\ P(X_{ijr}=x|\theta^{\left(X\right)},{Z_{ijr}}^{\left(X\right)},b_{i}) & =I\left(G_{ijr}=1\right)\left(F\left(d_{12}\right)-F\left(d_{11}\right)\right)+I\left(G_{ijr}=2\right)\left(F\left(d_{22}\right)-F\left(d_{21}\right)\right)\\ \text{logit}(P\left(G_{ijr}=1\right)) & =\xi_{r} \end{array}$$ (6)

where ${\underline{d}}_{1}=(d_{11},d_{12})$ is the censoring interval under the assumption that the data were rounded to the nearest 0.1 ha, and ${\underline{d}}_{2}$ is the censoring interval under coarsening with indicator *G*_*ijr*_ determining which interval to use, covariates recorded for each fire are denoted *Z*_*ijr*_^(*X*)^ with coefficient vector *β*^(*X*)^, and *ξ*_*r*_ is a constant probability of rounding within each region.

### 3.3 Estimation

The joint likelihood is calculated for each day as the likelihood for the hurdle component multiplied by the product of all of the likelihoods for each fire on that day. The conditional likelihood contributions from each submodel are shown in Eqs (1) and (4) and the joint likelihood is:

$$\begin{array}{cc} L_{i}(\theta^{\left(p\right)}, & \theta^{\left(N\right)},\theta^{\left(X\right)}\left|N_{ir}=n,X_{ijr}=x,b_{i}\right)=\\ & \prod_{r=1}^{R}[P\left(N_{ir}=n|\theta^{\left(p\right)},\theta^{\left(N\right)},b_{i}\right)\prod_{j=1}^{N_{ir}}P\left(X_{ijr}=x|\theta^{\left(X\right)},{Z_{ijr}}^{\left(X\right)},b_{i}\right)] \end{array}$$ (7)

The full likelihood is the integral of Eq (7) over all random effects:

$$\begin{array}{cc} L_{i}(\theta^{\left(p\right)}, & \theta^{\left(N\right)},\theta^{\left(X\right)}\left|N_{ir}=n,X_{ijr}=x\right)=\\ & \int_{B}L_{i}\left(\theta^{\left(p\right)},\theta^{\left(N\right)},\theta^{\left(X\right)}|N_{ir}=n,X_{ijr}=x,b_{i}\right)db \end{array}$$ (8)

where ℬ is the joint support of the random effects.

Estimation is carried out via Markov Chain Monte Carlo (MCMC) methods using a Gibbs sampler via the R interface to JAGS [39, 40]. To incorporate the truncated negative binomial likelihood into JAGS, the so-called 1s trick is employed, where the likelihood is estimated as *L*_*i*_(⋅) = *P*(*Z*_1_ = 1) and the likelihood of *Z*_1_ is a binomial distribution and *Z*_1_ is a vector of ones [37].

#### 3.3.1 Prior and hyperprior distributions

The prior distribution for the variance parameters of *b*_*i*_ and *X*_*ijr*_ were set as uniform on the interval (0, 20), based on the recommendations in [41]. The prior distributions for the coefficients were all chosen to be normal with mean 0 and standard deviation 5 (giving a precision of 1/25). The priors for the parameters in the spline terms were set according to [33]. The parameter associated with the first basis was set as a vague normal distribution (*a*_1_ ∼ *N*(*μ* = 0, *σ*^2^ = 100)). For the remaining parameters, a prior was set for the difference, i.e. *a*_*j*_ − *a*_*j*−1_ = *u*_*j*_ ∼ *N*(0, 100).

to be multivariate normal, with mean $\underline{0}$ and precision matrix (*A*^*T*^*A*)^−1/2^, where *A*_*ij*_ = 0.1 ∀ *i*, *j*. The hyperprior for the AR(1) parameter *ϕ* was set as uniform on the interval [−1, 1] to force the AR process to be stationary.

Shared hyperpriors are used for the region-specific intercepts *β*_*r*_ and λ_*r*_. In particular, *β*_*r*_ ∼ *N*(*β*_0_, precision = 1/25), where *β*_0_ ∼ *N*(0, 1/25) is the same mean for all regional parameters. This is equivalent to a random effects model for the intercepts of each region.

The prior distributions were also used for variable selection. This was performed by the inclusion of an indicator variable that forces a regression parameter to either be identically 0 or to follow a continuous posterior distribution. The indicator *I*(*β* = 0) follows a Bernoulli distribution, and the prior of the regression parameter is *I*(*β* = 0)*p*(*β*), where *p*(*β*) represents the unconstrained prior distribution. This is known as spike-and-slab regression, as was described by [42]. This method was chosen based on its simplicity, good mixing, and good separation properties as discussed in [43].

### 3.4 Goodness of fit and model comparison

#### 3.4.1 Predictions

Conditional on the random effects, predictions from the count distribution are obtained as the expectation of the truncated negative binomial distribution multiplied by the probability of a fire day. These marginal predictions can be used to evaluate the root mean squared error (RMSE) from the observed minus expected counts.

For the size distribution, predictions are not as straightforward because the true values are rounded. A potential approach is to round the predicted values and compare the observed minus expected number within each bin, but this does not account for the coarsening. We instead use a likelihood-based approach as described below.

#### 3.4.2 Widely Applicable Information Criterion

The Widely Applicable Information Criterion (WAIC, also known as Watanabe-Akaike Information Criterion, [44, 45]) is used to compare models. The calculation of the WAIC that we use is based on [46], and our notation in this section is identical to theirs.

The definition of WAIC is based on the log point wise predictive density (lppd), which is a concept closely related to leave-one-out cross validation.

$$\begin{array}{cc} \text{lppd} & =\text{log}\prod_{i=1}^{n}p_{\text{post}}\left(y_{i}\right)\\ \text{computed}\;\text{lppd} & =\sum_{i=1}^{n}\text{log}(\frac{1}{S}\sum_{s=1}^{S}p\left(y_{i}|\theta^{s}\right)) \end{array}$$

In the above equation, *θ*^*s*^ represents the *s*th draw from the posterior distribution out of *S* total draws, based on a MCMC algorithm. This formula calculates the average of the likelihood over all of the draws, then sums over the log of each term.

Next, we calculate the effective number of parameters (*p*_WAIC_) using the second definition in [46]. This quantity is used as a penalty for having too many unconstrained parameters.

$$\begin{array}{cc} p_{\text{WAIC2}} & =\sum_{i=1}^{n}{\text{var}}_{\text{post}}\left(\text{log}\left(p\left(y_{i}|\theta \right)\right)\right)\approx \text{effective}\;\text{number}\;\text{of}\;\text{parameters}\\ \text{computed}\;p_{\text{WAIC2}} & =\sum_{i=1}^{n}V_{s=1}^{S}\left(\text{log}\left(p\left(y_{i}|\theta^{s}\right)\right)\right) \end{array}$$

where the function $V_{s=1}^{S}(a)=\sum_{s=1}^{S}(a-\bar{a}{)}^{2}/(S-1)$ is the sample variance of the draws from the posterior distribution of the parameters.

Finally, the WAIC is calculated as follows:

$$\begin{array}{c} WAIC=-2(\text{lppd}-p_{\text{WAIC2}}) \end{array}$$ (9)

The WAIC was chosen because, as noted in [46], it is a more suited to the Bayesian approach in that it incorporates the entire posterior distribution rather than a point estimate.

### 3.5 Tests for independence

One of our primary goals is to quantify the dependence between the sizes and the counts. This can be done by testing whether the linking parameter is 0, whether the variance of the random effect is 0, or by comparing models with and without a linking parameter.

In this paper we test whether *γ*_*r*_ = 0. In this case, the count distribution retains a province-wide daily random effect and there is no information shared between the count and size models. Hence the count distribution is a mixed effects model. This is consistent with the findings of other authors such as [47] who found that a mixed effect framework was useful when modelling fire occurrence probability.

In Bayesian models, the posterior distribution is assumed to contain all of the information about a given parameter. For our test, we can look at whether *γ*_*r*_ = 0 is a credible value. We calculate a 95% Credible Interval (95%CI) and determine whether 0 is in the interval.

Other tests can be based on Bayes Factors, information criterion, or other calculations based on the posterior distribution as discussed in the Appendix. We note that the CI method is by far the simplest and performs just as well as the other tests with regards to the binary decisions in our simulations. Another advantage of this framework is that we are able to test for dependence within a single region. Tests based on model comparisons are not easily amenable to such situations.

## 4 Results

Models were fit separately to different years and for each of the two causes. For consistency across years, the optimal model was the one with the lowest WAIC for the most amount of years. For the lightning fires, this is a model with coarsening (M2), a spline for the hurdle component that is constant across the province, and an AR1 structure. The best models for person-caused fires also include M2 and an AR(1) structure, but had different spline terms for each region.

**Fig 3:**
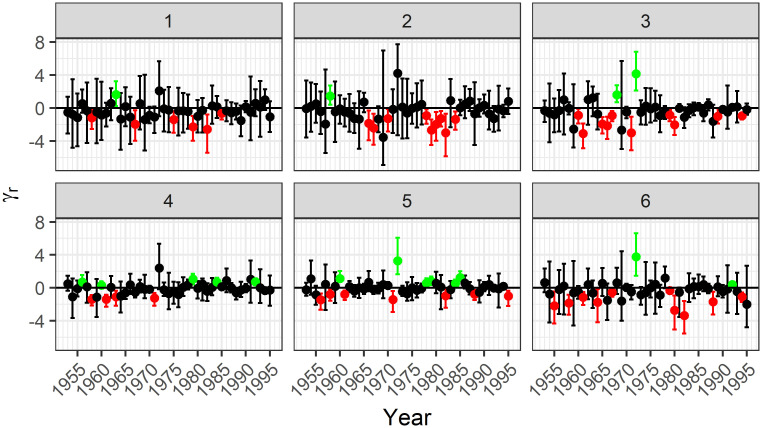
Posterior median estimates and 90% credible intervals for the linking parameter in the model for lightning-caused fires. There are very few years where the credible interval was entirely above 0, but several where it was below. This indicates that the size and count of lightning-caused fires often a negative association. Days with fewer fires had larger fires, and days with more fires had smaller fires.

**Fig 4:**
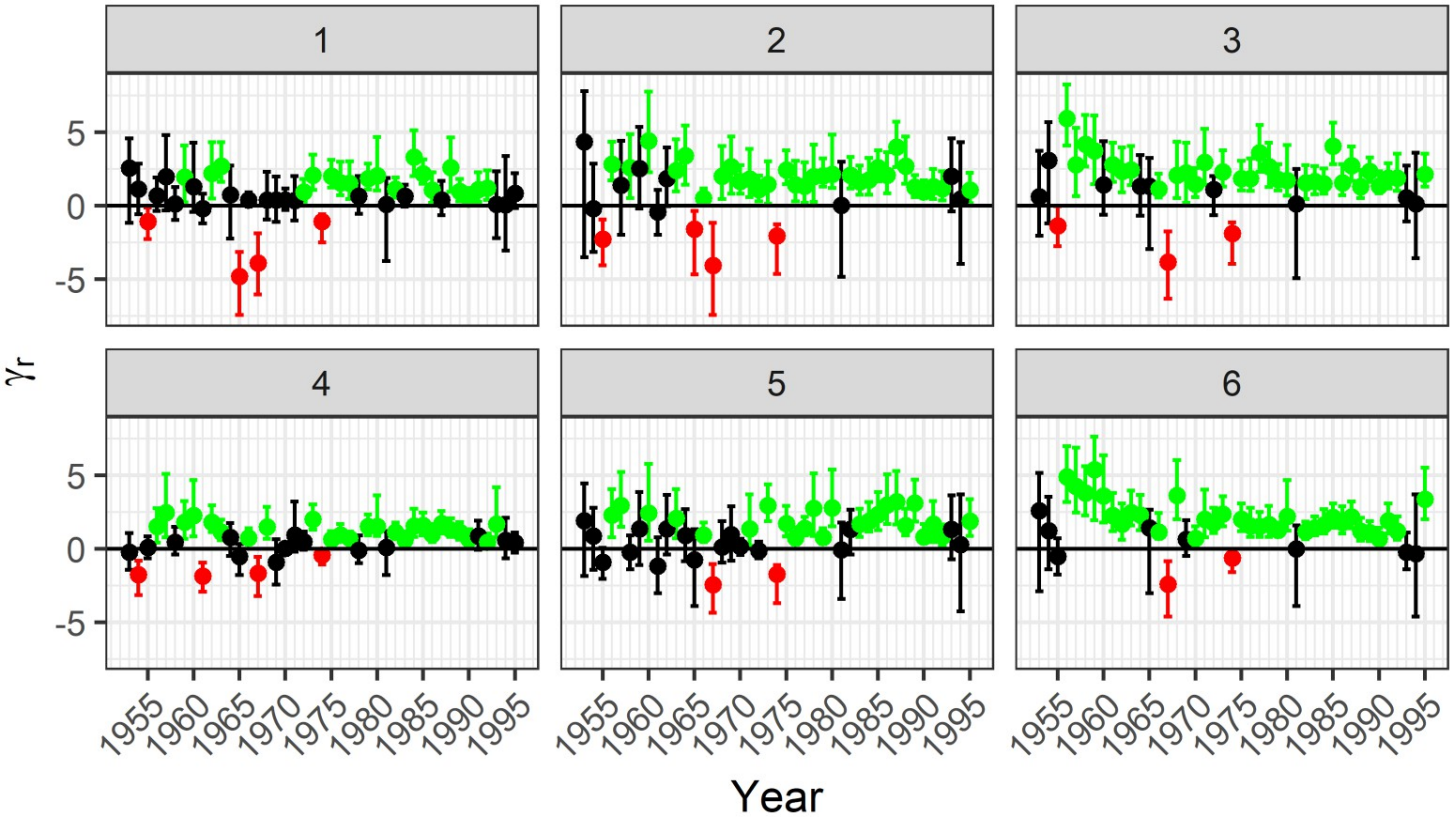
Posterior median estimates and 90% credible intervals for the linking parameter in the model for person-caused fires. In contrast to lightning-caused fires, person-caused fires tend to have positive association.

Figs 3 and 4 show the estimates for the linking parameter for each year along with a 95% CI for lightning- and person-caused fires, respectively. The points and intervals are coloured based on whether they are entirely above 0, entirely below 0, or contain 0.

For lightning-caused fires, the credible intervals that do not contain 0 are almost all negative, indicating that days with many fires tend to have smaller fires, and days with few fires tend to have larger fires. If there are many lightning-caused fires, then we expect that there are many days with lightning strikes and thus many rainy days. Multiple rainy days throughout the year would contribute to there being fewer fires. Since we included covariates that already account for the amount of rain on a single day, our model is capturing dependence on a larger time scale.

In contrast to lightning-caused fires, person-caused fires tend to have a positive association. If there are many person-caused fires, then this is likely due to an increase in human activity. This might be due to increased recreational activity or increased forestry activity, both of which would be associated with a lack of rain. Again our inclusion of weather covariates indicates that this observed dependence is either not based on the weather or occurs on a broader time scale.

**Fig 5:**
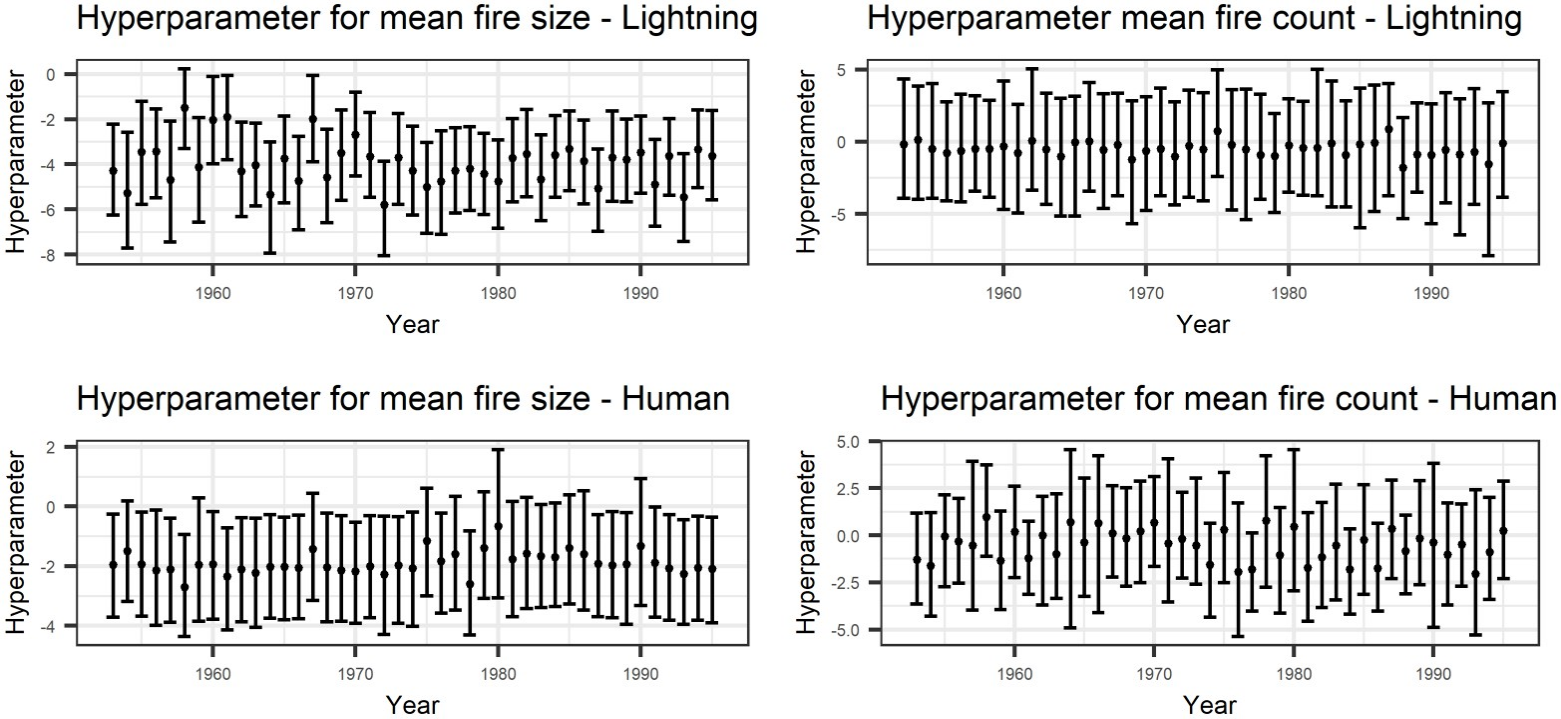
Hyperparameters for the mean of the size and count. By the construction of the model, the regional parameters are deviations from these values, with the hyperparameters representing the mean over all regions. All of these parameters are on the log scale.

Fig 5 shows the hyperparameters and their 95% CIs for both causes. These plots do not show any clear trend over time in the mean of either outcome. The values on the y-axis indicate that lightning-caused fires tend to be larger but there are fewer of them.

**Fig 6:**
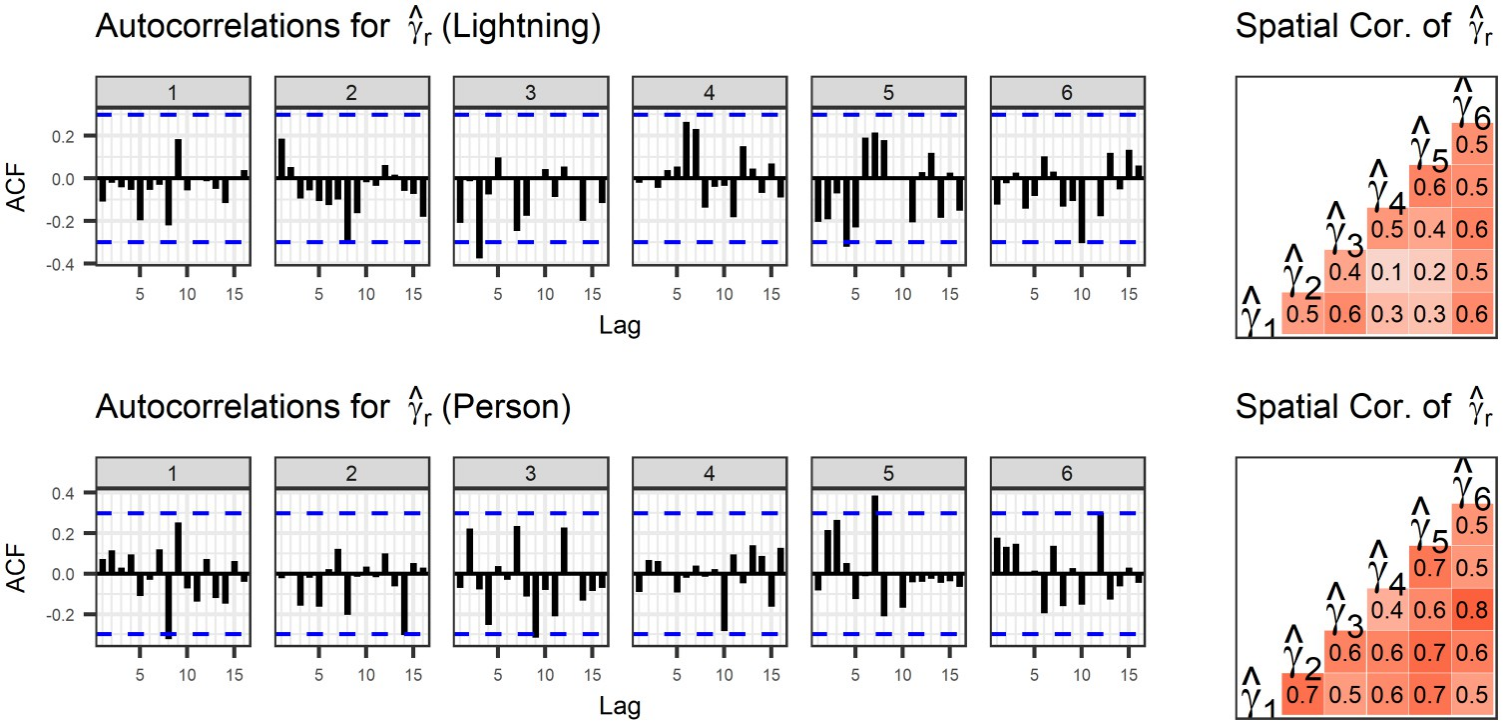
Correlation of the estimated linking parameter across space and time. The linking parameter in one year does not appear to predict the linking parameter in the next year. The linking parameter for lightning-caused fires is correlated for regions 1 and 2 (which are both interior from the coast and with similar forest cover) and regions 4 and 5 (which have no clear similarities). There is province-wide correlation among the linking parameters for person-caused fires.

Spatial and temporal correlations in the linking parameters are displayed in Fig 6. These correlations are calculated from the medians of the posterior distributions of the linking parameter. These correlations do not indicate any pattern over time for the linking parameter.

Estimated linking parameters for lightning-caused fires in regions 1 and 2 and regions 4 and 5 have the strongest correlations. Regions 1 and 2 are neighbours in a mountainous inland region, so their similarity is not surprising. Region 4 is a coastal region including Vancouver Island, while region 5 is a northern region to the east of the mountains.

For person-caused fires, the point estimates of *γ*_*r*_ in all of the regions appear to be strongly correlated. This indicates that if fire managers observe many large fires (or few small fires) in one region, they can expect something similar in another region. This only applies to human-caused fires, which may mean that this dependence is capturing broad weather trends or long weekends that lead to more (or less) human activity which has the possibility of igniting fires. This might manifest as increased recreational use or increased industrial activity, such as logging.

**Fig 7:**
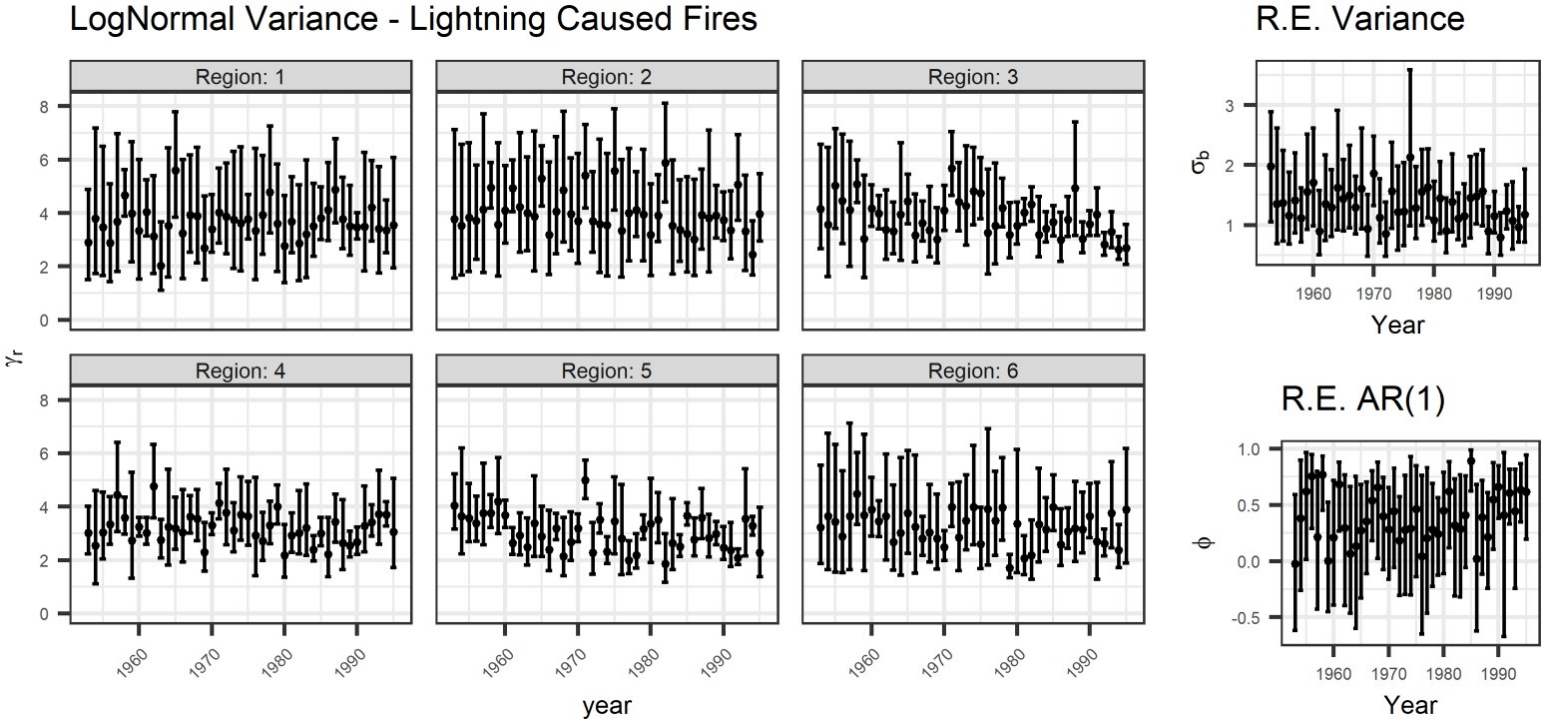
The variance parameters for lightning-caused fires. There is no apparent trend over time, except perhaps a slight decrease in region 5 and in the random effect variance, with an increase in the AR(1) parameter. The AR(1) parameter is constrained to be in the interval [-1,1].

**Fig 8:**
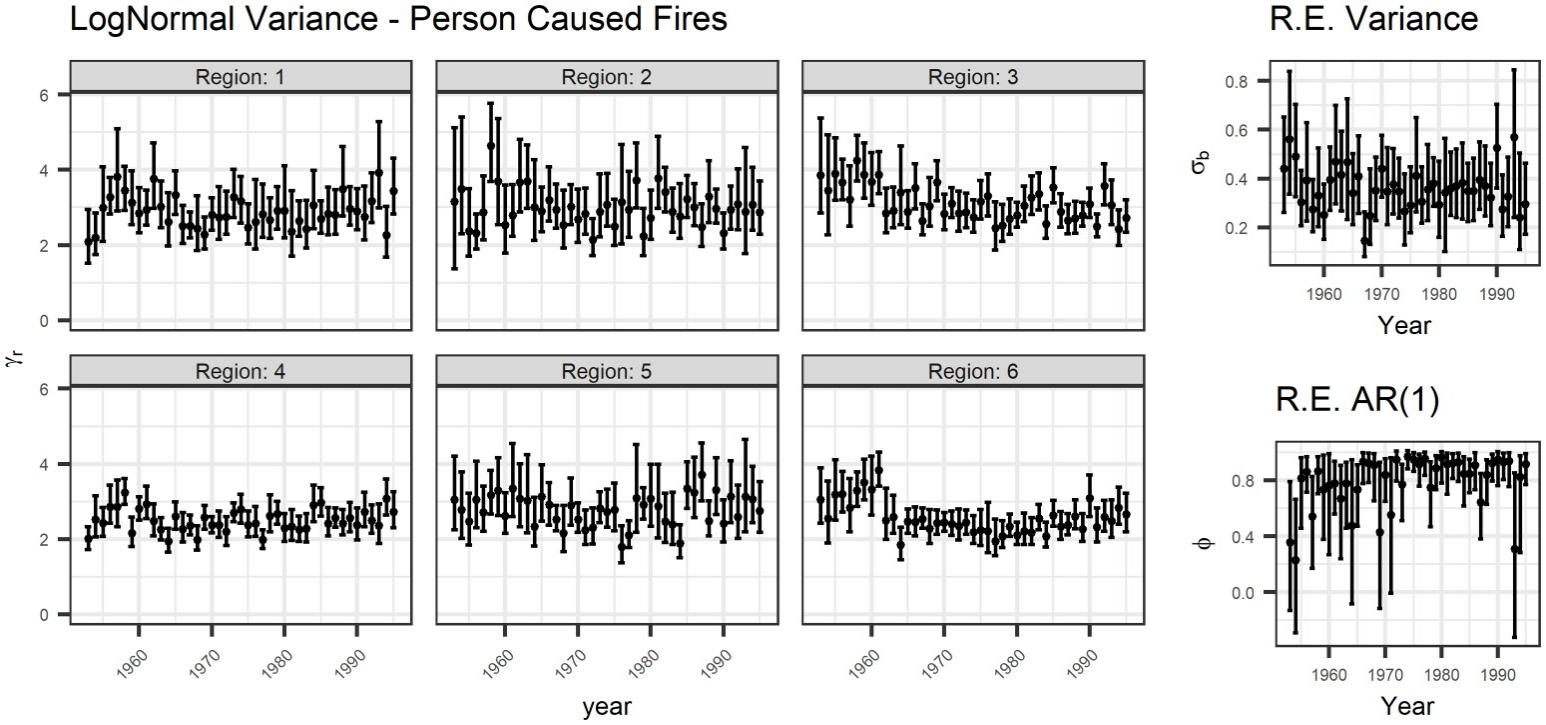
Variance parameters for person-caused fires. Again, there is a slight decrease in the estimates for region 5 and *σ*_*b*_ with an increase in the AR(1) parameter.

Figs 7 and 8 show the estimates of the variance parameters for lightning- and person-caused fires, respectively. Again, there is no clear pattern over time for lognormal variance *σ*_*r*_^2^ in the years in this study. However, the variance of the random effect *σ*_*b*_^2^ appears to be decreasing and the autoregressive parameter appears to be increasing. Since the random effect is shared between the models, this means that the variance of both models decreased, indicating that both the count and size of fires are less variable day-to-day. The increase in the autoregressive parameter *ϕ* also supports this conclusion.

Finally, note that the prior distribution for the autoregressive parameters is restricted to [-1, 1], meaning that the AR model is constrained to be stationary. The bottom right plot in Fig 8 (the variance plots for person-caused fires) demonstrates that this constraint may be too constrictive, and that the random effect is not stationary within each year.

## 5 Conclusions and discussion

We have introduced a model that allows for dependence between a zero-heavy count distribution and the summands of those counts. This was accomplished with a shared random effect, which is on the scale of the log of the number of fires and has a linking parameter in the size distribution. Such a framework could be employed in other contexts for jointly modelling the frequency and severity of events, such as insurance claims, hospital stays, or the number of herds along with the herd size.

One of the consequences of our model formulation is that the linking parameter provides a convenient foundation for a test of independence. For our data, the tests for independence concluded that there is indeed a form of independence between the number and the size of forest fires. The independence between these two is different depending on the cause of the fires. Lightning-caused fires tend to have negative dependence, whereas person-caused fires have positive dependence.

From the observed differences in direction of dependence among lightning- and person-caused fires, it appears that the dependence between size and count is driven by covariates that are either not observed or not observable. It is also possible that other physical properties are driving the dependence. Also note that person-caused fires are, on average, smaller than lightning-caused fires, and the dependence must be interpreted carefully.

We did not find any systematic changes in the parameter estimates over time. However, the data set presented in this paper only includes data up until 1995. Another data set is available with data up to 2015, but these data do not include covariate information. Analysis of the second data set has found several systematic patterns that are more prominent in 1995-2015. In particular, the lognormal variance parameters *σ*_*r*_ decrease while the random effect variance increases, indicating that the variance is better explained by the joint framework than the individual models alone. Neither data set includes 2017 or 2018, which were years with a large number of very large fires.

When *γ* = 0 the random effects term in the size distribution vanishes. This does not imply that there is no excess variability in the size distribution; it implies that any possible excess variability in the sizes is different from the excess variability in the counts. For models where *γ* = 0 it is still worthwhile to investigate a different day-specific random effect in each distribution. Incorporating this second random effect may or may not explain the observed pattern in the variance parameters; future work should investigate this.

A potential limitation to this study is that the linking parameter was constant over each year. We have investigated the use of spline terms for the linking parameter over the course of the fire season. According to the WAIC for each year, this adds too much complexity to the models. However, a future study might involve some other non-constant linking parameter, such as a piece-wise constant with carefully chosen time knots.

Another potential limitation is the size of the regions that we used. We assumed that they were approximately homogeneous, but regions 5 and 6 are much larger than the other regions (they are each approximately the size of the United Kingdon), so it is unlikely that these regions are truly homogeneous. The regions were chosen primarily for their homogeneity with respect to fire management efforts, but it may be useful to explore finer spatial partitions of the study region in future work, such as partitioning according to ecoregions (e.g., [48]). The definition of ecoregion should be chosen based on domain knowledge of wildland fire ecology.

Due to the spatial and temporal correlation found in this model, it may be useful to fire managers to have a version of this model that updates throughout the fire season to provide estimates of what type of year it is: positive dependence, negative dependence, or neutral. The AR structure in the random effect allows for prediction. If the linking parameter is negative and the random effects have been positive, fire managers could expect fewer fires but also be prepared for a large fire. To provide this to fire managers, the model would need to be deployed on a system that is easily accessed and understood.

## 6 Appendix A: Model selection “Power Study”

### 6.1 Simulation setup

In this appendix, we simulate from a simpler version of our model with varying degrees of separability and test which methods would lead to the correct conclusion. The simpler version of the model includes a single region and uses a simple negative binomial distribution for the count with no hurdle component and a constant mean and dispersion. The “true” sizes are lognormally distributed with a constant mean and variance.

$$\begin{array}{cc} N_{i} & ∼\text{NegBinom}(\lambda_{i}=\text{exp}\left(\lambda +b_{i}\right),\alpha )\text{;}\;\;i=1\ldots 215\\ {X_{ij}}^{*} & ∼\text{LogNormal}\left(\mu_{i}=\mu +\gamma b_{i},\sigma_{x}\right)\text{;}\;\;j=1\ldots N_{i}\\ X_{ij} & =\{\begin{array}{cc} \text{round}({X_{ij}}^{*},g_{1}) & \text{w.p.}\;p_{r}\\ \text{round}({X_{ij}}^{*},g_{2}) & \text{w.p.}\;1-p_{r} \end{array}\\ b_{i} & ∼N(0,\sigma_{b}) \end{array}$$

We considered parameters based on our estimates above, with λ = (−1, −0.5, 0), *σ*_*b*_ = (0.25, 0.5, 1), *σ*_*x*_ = 1, and *α* = 0.8. Our simulated data were rounded according to our assumed data generating process for our data, with a certain proportion of fires being rounded to the nearest 0.1 (represented by round(*X*_ij_*)) or to 0, 0.1, 0.5, 1, 1.5, or 2 (*g*_2_). To determine the power, we looked at values of *γ* from 0 to 1. We simulated 100 data sets from each combination of parameters.

### 6.2 Bayesian model selection

The most popular method of model selection is the Bayes Factor (BF), which is similar to the likelihood ratio in frequentist statistics. The BF is calculated as the ratio of the marginal likelihoods:

$$\begin{array}{c} BF_{01}=\frac{p(y|M_{0})}{p(y|M_{1})}=\frac{\int_{\Theta_{0}}p\left(y|\theta_{0},M_{0}\right)\pi_{0}\left(\theta_{0}\right)d\theta_{0}}{\int_{\Theta_{1}}p\left(y|\theta_{1},M_{1}\right)\pi_{1}\left(\theta_{1}\right)d\theta_{1}} \end{array}$$ (10)

In Eq (10), *π*_0_(*θ*_0_) and *π*_1_(*θ*_1_) are the prior distributions for parameter vectors *θ*_0_ and *θ*_1_, respectively. The BF does not rely on the posterior estimates of the data.

The BF does not exist in closed form for our model. There are several approximations to the marginal likelihoods that we investigated. A common approximation is the Harmonic Mean, which is shown in Eq (11). This approximation relies on posterior samples of the likelihood rather than prior samples. The harmonic mean estimate is criticised as having an infinite variance.

$$\begin{array}{c} \int_{\Theta_{1}}p\left(y|\theta \right)\pi \left(\theta \right)d\theta =E_{\pi (\theta )}\left[p\left(y|\theta \right)\right]\approx {\left[\frac{1}{S}\sum_{s=1}^{S}\frac{1}{p(y|\theta^{\left(s\right)})}\right]}^{-1}=HM_{01} \end{array}$$ (11)

In Eq (11), *S* represents the number of samples from the posterior distribution and *θ*^(*s*)^ is the *s*th sample of the parameter vector *θ* from a Gibbs sampling algorithm.

The harmonic mean is used to avoid numerical instability. The values of the likelihood tend to be too small for a computer to represent, whereas the inverse is not. If the numbers are not too small, one can simply look at the mean of the posterior samples of the likelihood. If the numbers are too large, the logarithm of the likelihood can be used.

In applying Eq (11), it was found that the likelihood values were still too small. To deal with this, we exploit the fact that the BF is a ratio. Adding a constant to each of log likelihood contributions does not change the ratio, so we subract the largest log likelihood value.

$$p(y|{\hat{\theta }}_{j})=\prod_{i=1}^{N}\left(p\left(y_{i}|{\hat{\theta }}_{j}\right)\right)\text{;}\;\;j=1,2$$ (12)

$$a=\text{max}\{\underset{i}{\text{max}}\left[\text{log}\left(p\left(y_{i}|{\hat{\theta }}_{0}\right)\right)\right],\underset{i}{\text{max}}\left[\text{log}\left(p\left(y_{i}|{\hat{\theta }}_{1}\right)\right)\right]\}$$ (13)

$$\frac{E(\sum_{i=1}^{N}\text{log}\left(p\left(y_{i}|{\hat{\theta }}_{0}\right)-a\right))}{E(\sum_{i=1}^{N}\text{log}\left(p\left(y_{i}|{\hat{\theta }}_{1}\right)-a\right))}=\frac{\text{exp}(a^{N})}{\text{exp}(a^{N})}BF_{01}=BF_{01}$$ (14)

This identity greatly improved the stability of the Bayes Factors computation.

Another estimate is the Naive Bayes Estimator. This is the estimator that arises naturally from the first two expressions in Eq (11), where we are interested in the expectation of the likelihood with respect to the prior distributions. The primary issue with this estimate is numerical stability. The draws from the prior distribution will often be in areas of low density for the likelihood, so there will be many values that are indistinguishable from 0. We included this estimate in our analysis, but the numerical instability is apparent in the results.

The final estimate of the BF that we consider is known as the Savage-Dickey Density Ratio (SDDR). The SDDR is only applicable in tests where $M_{0}$ is a point density and $M_{1}$ is a distribution (e.g. *γ* = 0 versus *γ* ∼ *π*(*γ*)). The SDDR for testing a parameter equal to 0 is calculated as follows:

$$\begin{array}{c} SDDR_{01}=\frac{\pi (0)}{\pi \left(\hat{\theta }\right){|}_{\hat{\theta }=0}} \end{array}$$ (15)

This is simply the ratio of the value of the prior distribution at 0 divided by the value of the posterior distribution at 0.

For all of the BF estimates, we convert the Bayes Factor into a binary decision by treating any BF larger than 3 as evidence against *M*_0_. This value was chosen based on hueristic guidelines from [49], who define a BF between 3 and 10 to be “moderate evidence.”

Other model selection methods that do not invlolve Bayes Factors are also investigated. The Bayesian framework allows for probabilistic assessments of the parameters in the model, so several frameworks for testing point-Null hypotheses have been proposed. We employ two in particular, as implemented in the bayestestR R package [50].

The first is the Probability of Direction (pd), introduced in [51]. This is defined as the integral of the posterior distribution from 0 to either positive or negative ∞, depending on the sign of the median of the posterior.

The second is the Region of Practical Equivalence (ROPE) test. This test sets up a region around the null value (0 in this case) that would be considered “practically equivalent,” i.e. close enough to 0 to be indistinguishable. Usually, this region is 0 plus or minus one standard deviation of the posterior distribution. The ROPE test reports the proportion of the Highest Density Interval that lines within the ROPE.

In both tests, a cutoff point must be chosen. To be consistent with previous studies, we use a 5% cutoff value. This is approximately equivalent to a p-value. We note that there is no equivalence between p-values and Bayes Factors (see, e.g. [52]).

Similar to [53], we also employ a binary test based on the WAIC. For this study, we simply compare the difference in WAIC between the full model and a model with *γ* constrained to be 0. Since WAIC is fully Bayesian, the full posterior distributions of the WAIC in each model could be compared. However, we have not encountered a principled approach to such a comparison, so we simply look at the difference in WAIC.

The variable selection method that we employed for the covariates can also be applied as a fully Bayesian test of a point null hypothesis. The posterior can be constrained to 0 with some probability, and this probability can be a parameter in the model. The probability that the posterior is identically 0 works as a hypothesis test. Again, we use the 5% cutoff.

The final test that we consider is the one that we used in this paper. The posterior distribution contains all of the information about the parameter, so simply constructing a credible interval and checking whether 0 is in that interval should be sufficient. Again, we use a 5% cutoff as a binary test.

### 6.3 Power study results

**Fig 9:**
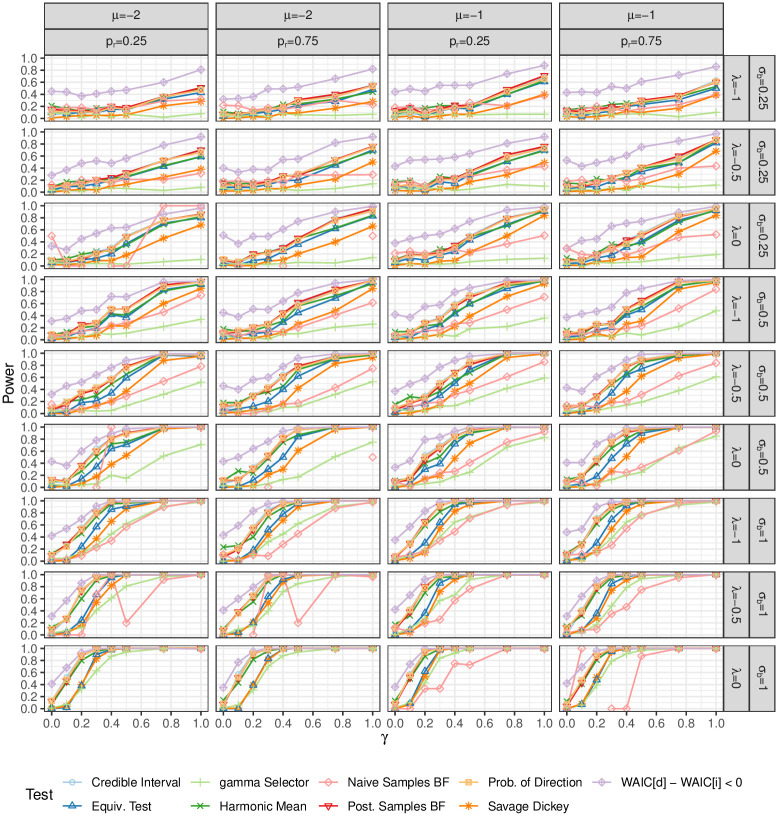
Results of the power study. The credible interval test and the Bayes factors that relied on posterior samples performed the best overall. Simply looking at whether the WAIC is smaller has high power at the expense of high type 1 error. The Savage Dickey representation of the BF might have higher power with better prior distributions. Using the variable selection method on *γ* has very low power, likely because *γ* is the coefficient of a latent variable rather than an observed covariate. The equivalence test and probability of direction have slightly lower power than simply checking the credible interval.

The results of this power study are shown in Fig 9. Unsurprisingly, all of the methods performed better when there was more data available (i.e. with larger λ, there are more observed *X*_*ij*_*s*). The tests also had more power when *σ*_*b*_ was larger. Low values of *σ*_*b*_ also represent a small effect size which explains the low power in all of the tests.

The estimates of the BF based on posterior draws performed quite well, and the credible interval and pd tests were approximately equivalent. Since the credible interval test is easily calculated, this test is preferred.

The pd is also easy to calculate and provide a one-sided test where the direction is chosen based the posterior. In the frequentist framework, this concept would be troublesome as it is choosing the direction of the test based on which one gives a smaller p-value. However, the Bayesian posterior distribution does not have the same interpretation and thus avoids the issue of testing multiple hypotheses in search of a significant result (sometimes referred to as p-hacking).

As is expected, the power of the WAIC test is approximately 50% when *γ* is constrained to 0. In light of this, the power results are not as impressive as they seem.

The numerical instability of the BF estimate based on prior samples is obvious. In some cases, there were no BF estimates that had non-zero marginal probabilities, and so there is no estimate of the power.

The SDDR has the lowest power out of all of the Bayes Factor estimates. This is likely because the SDDR is very sensitive to the choice of prior.

The variable selection method also performed quite poorly. This is likely because of the extra variation introduced by the latent variable which would not be present in observed covariates.
